# Different Immune Responses of Hemocytes from *V. parahaemolyticus*-Resistant and -Susceptible Shrimp at Early Infection Stage

**DOI:** 10.3390/biology13050300

**Published:** 2024-04-26

**Authors:** Wenran Du, Shihao Li, Fuhua Li

**Affiliations:** 1 School of Marine Science and Engineering, Qingdao Agricultural University, Qingdao 266109, China; duwenran@qdio.ac.cn; 2 CAS and Shandong Province Key Laboratory of Experimental Marine Biology, Institute of Oceanology, Chinese Academy of Sciences, Qingdao 266071, China; fhli@qdio.ac.cn; 3 Laboratory for Marine Biology and Biotechnology, Qingdao Marine Science and Technology Center, Qingdao 266237, China; 4 Key Laboratory of Breeding Biotechnology and Sustainable Aquaculture, Chinese Academy of Sciences, Wuhan 430072, China; 5 Center for Ocean Mega-Science, Chinese Academy of Sciences, Qingdao 266071, China

**Keywords:** penaeid shrimp, hemocytes, *Vibrio parahaemolyticus*, glycolysis, immunity

## Abstract

**Simple Summary:**

Identification of differentially expressed genes (DEGs) between disease-resistant and -susceptible animals is a method to screen genes contributing to anti-disease. Previously, we found that there were few genes differentially expressed in hemocytes between *V. parahaemolyticus*-resistant and -susceptible shrimp. The present study hypothesized that these shrimp had different immune responses against pathogen infection and tested it using comparative transcriptome analysis.

**Abstract:**

*Vibrio parahaemolyticus* is one of the main causative agents leading to acute hepatopancreatic necrosis disease, the severe bacterial disease that occurs during shrimp aquaculture. Hemocytes play important roles during *Vibrio* infection. Previously, we found that there were few differentially expressed genes (DEGs) between hemocytes from *V. parahaemolyticus*-resistant and -susceptible shrimp before infection. We considered that there should be different immune responses between them after a pathogen infection. Here, the transcriptome data of hemocytes from *V. parahaemolyticus*-resistant and -susceptible shrimp before and after a pathogen infection were compared. The results showed that there were 157 DEGs responsive to infection in *V. parahaemolyticus*-resistant shrimp, while 33 DEGs in *V. parahaemolyticus*-susceptible shrimp. DEGs in *V. parahaemolyticus*-resistant shrimp were mainly related to immune and glycolytic processes, while those in *V. parahaemolyticus*-susceptible shrimp were mainly related to metabolism, with only two DEGs in common. A further analysis of genes involved in glucose metabolism revealed that GLUT2, HK, FBP, and PCK1 were lowly expressed while PC were highly expressed in hemocytes of the *V. parahaemolyticus*-resistant shrimp, indicating that glucose metabolism in shrimp hemocytes was related to a *V. parahaemolyticus* infection. After the knockdown of PC, the expression of genes in Toll and IMD signaling pathways were down-regulated, indicating that glucose metabolism might function through regulating host immunity during *V. parahaemolyticus* infection. The results suggest that the immune responses between *V. parahaemolyticus*-resistant and -susceptible shrimp were apparently different, which probably contribute to their different *V. parahaemolyticus* resistance abilities.

## 1. Introduction

The frequent outbreak of shrimp diseases leads to a large amount of production losses in global shrimp culture, which seriously affects its economic value. The sustainable development of shrimp culture is closely related to the control of shrimp diseases. In addition to the pathogen, the disease resistance of the host is also an important factor in disease control [1,2]. The acute hepatopancreatic necrosis disease (AHPND), a disease caused by bacteria [3], can lead to serious mortality of cultured shrimp and cause substantial economic losses to the shrimp culture industry. *Vibrio parahaemolyticus*, one of the main pathogens of AHPND, carries an extrachromosomal plasmid that encodes a binary toxin PirA^Vp^ and PirB^Vp^, homologous to the Photorhabdus insect-related (Pir) toxins [4]. These toxins have been identified as the primary contributors to AHPND toxicity [5].

Understanding the mechanisms of disease resistance is an important theoretical basis to solve the problem of shrimp disease. At present, a large number of genes or regulatory processes related to shrimp disease resistance have been reported. In *Litopenaeus vannamei*, a comparison of gene expression in cephalothoraxes of shrimp between resistant and susceptible families against VP_AHPND_ revealed that immune response and energy metabolism might contribute to the resistance of shrimp against VP_AHPND_ [6]. A comparative transcriptome of gills from shrimp with varying resistances against VP_AHPND_ revealed that some genes participating in endocytosis, protein synthesis and immune responses were related to *Vibrio*-resistance of *L. vannamei* [7]. Comparative transcriptome analysis of the hepatopancreas from shrimp in two families with different resistances to VP_AHPND_ revealed that some immune-related genes were highly expressed in shrimp from the resistant family while metabolic-related genes were highly expressed in shrimp from the susceptible family [8]. An integrated analysis of the hepatopancreas between transcriptome and metabolome in *L. vannamei* suggested that the relative prosperous metabolic state in Vp_AHPND_-susceptible shrimp might benefit bacterial proliferation, while the activation of NF-κB and cAMP pathways induced by arachidonic acid metabolism in Vp_AHPND_-resistant shrimp after infection might contribute to host resistance ability [9]. These studies revealed that metabolism and immunity in shrimp jointly participated in the defense process against pathogen infection.

Hemocytes play an important role in the immune defense system of shrimp, not only as the executors of the cellular immune process but also for the synthesis and release of a variety of immune factors, providing a material basis for humoral immunity [10]. Previously, we established a method to extract hemocytes from uninfected shrimp with different resistances to *V. parahaemolyticus*. Transcriptome analysis found that several genes were differentially expressed in hemocytes between *V. parahaemolyticus*-resistant and -susceptible shrimp at the background level [11], leading to a speculation that the differences in shrimp resistance ability against *V. parahaemolyticus* infection might be attributed to the variations in their immune responses. In the present study, shrimp from the same batch as previously reported [11] were used to analyze whether there were different immune responses between hemocytes from *V. parahaemolyticus*-resistant and -susceptible shrimp after pathogen infection. Hemocytes were collected from shrimp before infection and at three hours post-infection, and comparative transcriptome analysis was conducted to identify genes related to immune responses in *V. parahaemolyticus*-resistant and -susceptible shrimp, respectively. The data showed apparently different immune responses between them, which might contribute to the variation in their resistance abilities against *V. parahaemolyticus* infection.

## 2. Materials and Methods

### 2.1. Experimental Animals

The experimental animals *Litopenaeus vannamei* that were from the same batch of shrimp, population 2, in our previously study [11], were acquired from Rizhao breeding farm in Shandong and cultured in aerated seawater at 25 °C. Shrimp used in the experiment weighed 19.5 ± 1 g. Before the experiments, shrimp were confirmed to be free of *Vibrio* pathogens as described in our previous study [11].

### 2.2. Experiment Design and Sampling

**Figure 1:**
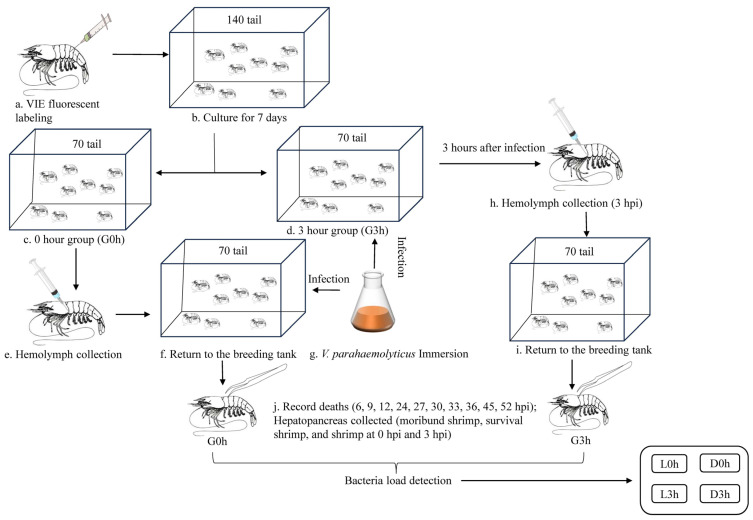
Workflow of the experimental design.

A total of 140 individuals were used for immune challenge. The experiment design workflow was illustrated in Figure 1. Each individual was labeled with vibration-induced emission (VIE) through injecting into the third segment and continued to be temporarily cultured in the aerated seawater at 25 ± 1 °C for seven days and fed twice a day. Two groups, group 0 h (G0h) and group 3 h (G3h), with 70 individuals each, were set. For G0h, 300 μL hemolymph was extracted from each individual with a sterile syringe containing equal volume of ice-cold anticoagulant containing 27 mM trisodium citrate, 336 mM sodium, 115 mM glucose, and 9 mM EDTA·Na_2_·2H_2_O, at pH 7.4. Then, the shrimp were infected with *V. parahaemolyticus* under immersion infection with a final concentration of 5 × 10^6^ CFU/mL. The infection experiments were performed using strains isolated from our laboratory [12]. For G3h, the shrimp were directly infected under the same condition. At 3 h post-infection (hpi), 300 μL hemolymph was extracted from each individual and then put back for immersion infection. The hemolymph was centrifuged at 4 °C and at 1000× *g* for 10 min. The supernatant was removed, and the hemocytes were frozen in liquid nitrogen and stored at −80 °C. The mortality was recorded at 6, 9, 12, 21, 24, 27, 30, 33, 36, 45, and 52 hpi. The hepatopancreases of moribund shrimp at each time point (D0h and D3h), shrimp before infection (0 hpi) and 3 h post-infection (3 hpi), and survival shrimp after 52 hpi (L0h and L3h) were collected for *Vibrio* load detection.

### 2.3. DNA Extraction and Bacteria Load Detection

Genomic DNA was extracted from the above collected the hepatopancreas using the Genomic DNA Extraction Kit following the instructions (TIANGEN, Beijing, China). NanoDrop 2000 (Thermo Fisher Scientific, Waltham, MA, USA) was used to measure the concentration and purity of DNA, and 1% agarose gel electrophoresis was used to test the integrity of DNA. PirA^Vp^ copy numbers in extracted DNA were detected by TaqMan probe fluorescent quantitative PCR using Ependorf MasterCycler EP Realplex (Ependorff, Hamburg, Germany), and each assay was carried out in four replicates. Primers and procedures used for RT-qPCR are shown in Table S1.

### 2.4. RNA Extraction and Transcriptome Sequencing

According to the survival data and *Vibrio* loads, shrimp were divided into *V. parahaemolyticus*-resistant and -susceptible individuals. Hemocytes from *V. parahaemolyticus*-resistant and -susceptible shrimp in G0h and G3h groups were mixed, respectively. In G0h, the samples were named as L0h for *V. parahaemolyticus*-resistant shrimp and D0h for *V. parahaemolyticus*-susceptible shrimp. In G3h, the samples were named as L3h for *V. parahaemolyticus*-resistant shrimp and D3h for *V. parahaemolyticus*-susceptible shrimp. Each sample included three replicates. Total RNA was extracted from each sample with RNAiso Plus (TaKaRa, Kyoto, Japan) according to the manufacturer’s instructions. The concentration, purity, and integrity of the total RNA was measured by NanoDrop 2000 (Thermo Fisher Scientific, Waltham, MA, USA) and 1% agarose gel electrophoresis, respectively. About 1 μg total rRNA from each sample was pre-treated with gDNA Eraser (TaKaRa, Kyoto, Japan), and the mRNA was enriched with a conventional kit (New England Biolabs, Ipswich, MA, USA). The mRNA was fragmented and used as template to synthesize the first-strand cDNA using random hexamers. The second-strand cDNA was synthesized after adding dNTPs, RNaseH, and DNA polymerase I. The purified double-stranded cDNA was repaired at the end, and the ploy(A) was added to connect the Illumina sequencing adapter. Finally, the sequencing was performed using the Illumina HiSeq2500 platform at Guangzhou Gene Denovo Biotech Co., Ltd. (Guangzhou, China).

### 2.5. Reads Mapping and Annotation

Considering the quality of the data, only reads with a mass fraction greater than 10% were used for further analysis. Clean reads were mapped to the reference genome [13] using HISAT2.2.4 [14]. The mapped reads were assembled with StringTie v1.3.1 [15,16] in a reference-based approach. The reconstructed transcripts were compared to the reference genome, and new genes were annotated functionally by comparison with nucleotide sequences (NTs), non-redundant protein sequences (NRs), Swiss-Prot, protein orthologous clusters (COG), and the Kyoto Encyclopedia of Genes and Genomes (KEGG) databases.

### 2.6. Differential Expression and Enrichment Analysis

The expression level of each transcript was determined using its FPKM (fragments per million mapped reads per thousand base transcripts) values. DEGs were analyzed using the edgeR software package (http://www.rproject.org/, accessed on 13 June 2023) with a false discovery rate (FDR) of <0.05 and the absolute multiple change of ≥2. In addition, a correlation analysis among samples was performed to confirm the reliability of the data. GO function and KEGG pathway enrichment analysis of DEGs were carried out using the online OmicShare tool (http://www.omicshare.com/tools, accessed on 15 June 2023). Statistical significance was confirmed with Q values of <0.05.

### 2.7. RNA Interference

Primers with T7 promoter sequences were designed to amplify the DNA template of EGFP and LvPC for dsRNA synthesis. The PCR programs were performed according to the product size and primer annealing temperature (Table S1). PCR products were detected by electrophoresis on a 1% agarose gel and purified with the MiniBEST DNA Fragment Purification Kit (TaKaRa, Kyoto, Japan). The dsRNA was synthesized using Transcriptaid T7 High Yield Transcription Kit (Thermo Fisher Scientific, Waltham, MA, USA) and purified using a mixture of phenol and chloroform. The concentration of dsRNA was checked using NanoDrop 2000, and the quality of dsRNA was detected using 1% agarose electrophoresis.

After dosage optimization, 2 μg/g shrimp was used for RNA interference. Hemocytes of five shrimp in each group were collected as one sample at 48 h after dsRNA interference. Three replicates were set for each treatment. Total RNA was extracted according to Section 2.4.

### 2.8. Quantitative Real-Time PCR

About 1 μg total RNA was used to synthesize the first strand of cDNA with the PrimeScript™ RT kit with gDNA Eraser (TaKaRa, Kyoto, Japan). The product was diluted 20 times using nuclease-free water. qRT-PCR was carried out with THUNDERBIRD^®^ SYBR^®^ qPCR Mix in Eppendorf Mastercycler ep realplex (Eppendorf, Hamburg, Germany) at a total volume of 10 μL (5 μL qPCR mixture, 1 μL diluted cDNA, 0.3 μL forward and reverse primers, and 3.4 μL nuclease-free water). The amplification program was set as follows: 95 °C for 2 min; 40 cycles of 95 °C for 15 s, annealing temperature for 15 s and 72 °C for 30 s; and followed by a melting curve. Each sample was assessed in three technical replicates. In order to standardize expression levels, 18S rRNA was used as an internal control. The expression profile of each gene was calculated by 2^−∆∆CT^ and shown with log2 fold change values. Primers used for the detection of genes in Toll and IMD pathways are listed in Table S1.

### 2.9. Statistical Analysis

All data were displayed in the form of mean ± S.E. and calculated using one-way analysis of variance (ANOVA) and Ducan’s Multiple Comparisons. Differences between comparisons were considered statistically significant at *p* < 0.05 and statistically highly significant at *p* < 0.001.

## 3. Results

### 3.1. The Loads of V. parahaemolyticus in Hepatopancreas of Treated Shrimp

**Figure 2:**
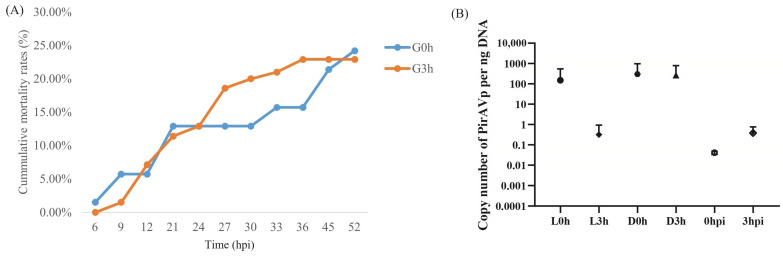
The cumulative mortality rates and the load of *V. parahaemolyticus* in the hepatopancreas of infected shrimp from two groups, G0h and G3h. (**A**) shows the accumulative mortality rates of shrimp in two groups. (**B**) shows the copy number of *V. parahaemolyticus* per ng hepatopancreatic DNA in different samples. L0h, the sample of *V. parahaemolyticus*-resistant shrimp in G0h. L3h, the sample of *V. parahaemolyticus*-resistant shrimp in G3h. D0h, the sample of *V. parahaemolyticus*-susceptible shrimp in G0h. D3h, the sample of *V. parahaemolyticus*-susceptible shrimp in G3h. 0 hpi, the sample of shrimp before *V. parahaemolyticus* infection. 3 hpi, the sample of shrimp at 3 h post *V. parahaemolyticus* infection.

The cumulative mortality rates for two shrimp groups G0h and G3h were 24.2% and 22.9%, respectively (Figure 2A). The standard curve equation for PirA^Vp^ was y = 3.0362x + 34.723, where x was the logarithm value of the *Vibrio* copy number per μL standard plasmid, and y was the Ct value. The correlation coefficient R^2^ = 0.9263, which was a good linear relationship between the concentration of the standard sample and the Ct value. As shown in Figure 2B, the loads of *V. parahaemolyticus* in hepatopancreas of shrimp in L0h, D0h, and D3h were 151, 302, and 268 copy per ng hepatopancreatic DNA, respectively, while those in L3h, 0 hpi, and 3 hpi were very low, which were less than one copy per ng hepatopancreatic DNA. The data suggest that *V. parahaemolyticus* did not proliferate substantially at 3 hpi.

### 3.2. Differential Immune Responses of Hemocytes against Pathogen Infection between V. parahaemolyticus-Resistant and -Susceptible Shrimp

**Figure 3:**
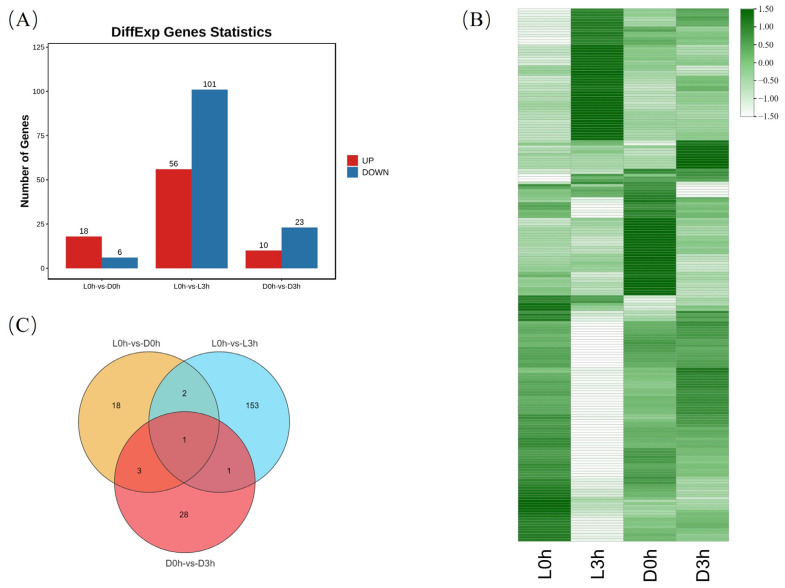
Differentially expressed genes (DEGs) from L0h vs. D0h comparison, L0h vs. L3h comparison, and D0h vs. D3h comparison. A total of 212 DEGs, including 24 DEGs from L0h vs. D0h comparison, 157 DEGs from L0h vs. L3h comparison, and 33 DEGs from D0h vs. D3h comparison, were identified and displayed in the histogram (**A**), the heatmap (**B**), and the Venn diagram (**C**).

Differential analysis identified 24 differentially expressed genes (DEGs) between L0h and D0h, including 18 up-regulated DEGs and 6 down-regulated DEGs in D0h, 157 DEGs between L0h and L3h, including 56 up-regulated DEGs and 101 down-regulated DEGs in L3h, and 33 DEGs between D0h and D3h, including 10 up-regulated DEGs and 23 down-regulated DEGs in D3h (Figure 3A,B, Table S2). Among the DEGs from two groups, only five genes were shared by at least two comparisons. Particularly, only two genes were shared by L0h vs. L3h comparison and D0h vs. D3h comparison, including aromatic-L-amino-acid decarboxylase-like gene and transmembrane protease serine 9-like gene (Figure 3C, Table S2), suggesting quite different immune responses of hemocytes against pathogen infection between *V. parahaemolyticus*-resistant and -susceptible shrimp.

**Figure 4:**
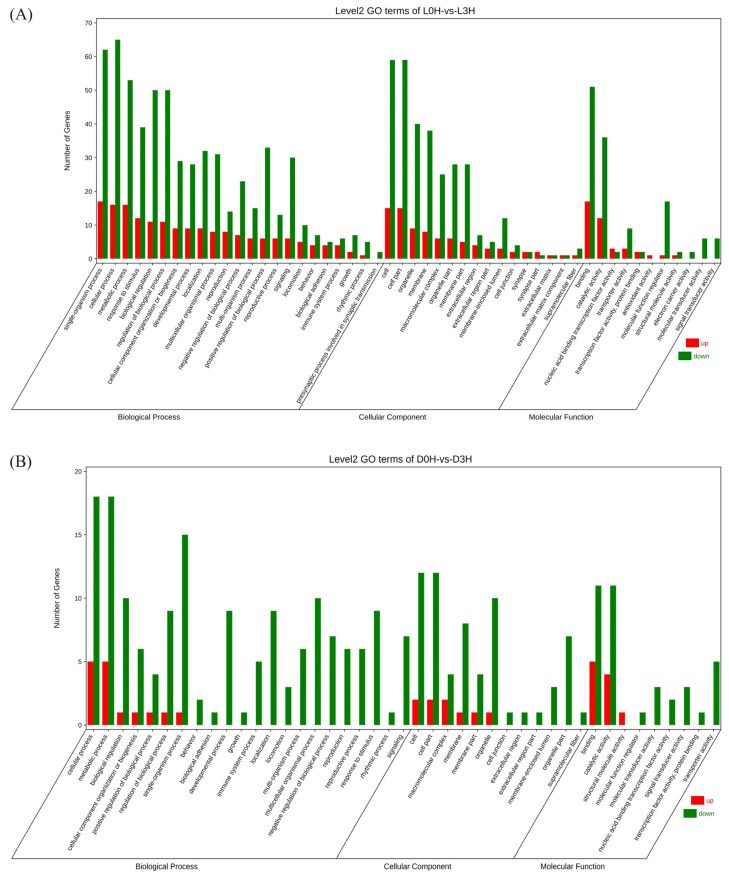
Gene ontology (GO) enrichment analysis of DEGs from L0h vs. L3h comparison (**A**) and D0h vs. D3h comparison (**B**).

**Figure 5:**
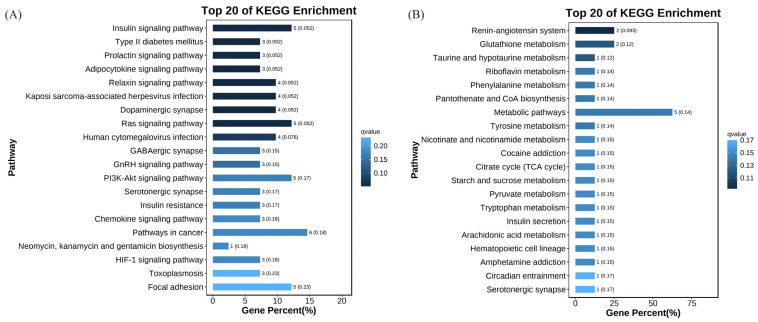
KEGG enrichment analysis of DEGs from L0h vs. L3h comparison (**A**) and D0h vs. D3h comparison (**B**).

Gene ontology (GO) analysis showed that DEGs from L0h vs. L3h comparison were mainly enriched in the biological processes including cellular processes, single organism processes, metabolic processes, biological processes, biological process regulation, and stimulus response processes. In the molecular function category, most DEGs were involved in binding and catalytic activity. Among cellular components, most DEGs were enriched in cells, cell parts, organelles, and membranes. DEGs from D0h vs. D3h comparison were mainly enriched in the biological processes including metabolic processes, single organism processes, biological regulation, biological process regulation, and multicellular biological processes. In the molecular function category, most DEGs were involved in binding and catalytic activity. Among cellular components, most DEGs were enriched in cells, cell parts, organelles, and membranes (Figure 4). KEGG analysis showed that no DEGs from either comparison were significantly enriched in signaling pathways (Figure 5).

### 3.3. The Glycolysis Process Showed Different Responses to Pathogen Infection in Hemocytes between V. parahaemolyticus-Resistant and -Susceptible Shrimp

**Figure 6:**
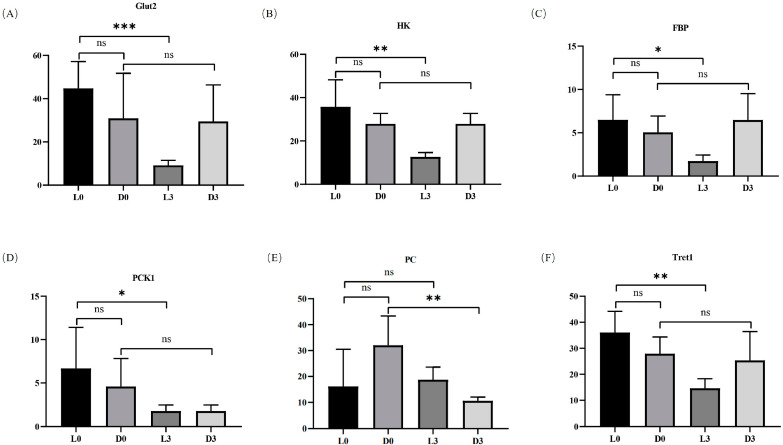
Expression profiles of several glucose metabolism-related genes in the hemocytes of *V. parahaemolyticus*-resistant and -susceptible shrimp. (**A**–**F**) show the expression of solute carrier family 2 promoting glucose transporter member 1-like isomer X1 (Glut2), hexokinase type 2-like (HK), 6-phosphofructo-2-kinase/fructose-2,6-bisphosphatase (FBP), phosphoenolpyruvate carboxykinase 1 (PCK1), and facilitated trehalose transporter (Tret1), respectively. L0h, the sample of *V. parahaemolyticus*-resistant shrimp in G0h. L3h, the sample of *V. parahaemolyticus*-resistant shrimp in G3h. D0h, the sample of *V. parahaemolyticus*-susceptible shrimp in G0h. D3h, the sample of *V. parahaemolyticus*-susceptible shrimp in G3h. Significant differences between PC knockdown and control groups are shown with an asterisk at *p* < 0.05, two asterisks at *p* < 0.01, and three asterisks at *p* < 0.001.

Although no pathway was significantly enriched for DEGs by KEGG analysis, functional annotation showed that several genes in the glycolysis process had apparently different expression patterns against pathogen infection in hemocytes between *V. parahaemolyticus*-resistant and -susceptible shrimp. In hemocytes of *V. parahaemolyticus*-resistant shrimp, solute carrier family 2 promoting glucose transporter member 1-like isomer X1 (Glut2), hexokinase type 2-like (HK), 6-phosphofructo-2-kinase/fructose-2,6-bisphosphatase (FBP), and phosphoenolpyruvate carboxykinase 1 (PCK1) were all down-regulated after pathogen infection (Figure 6A–D). In addition, the trehalose transporter (facilitated trehalose transporter, Tret1) was also down-regulated after pathogen infection in the hemocytes of *V. parahaemolyticus*-resistant shrimp (Figure 6F). However, the expression profiles of these genes were not apparently affected by pathogen infection in the hemocytes of *V. parahaemolyticus*-susceptible shrimp. Furthermore, the expression level of pyruvate carboxylase (PC) was only down-regulated after pathogen infection in the hemocytes of *V. parahaemolyticus*-susceptible shrimp (Figure 6E). The data suggested that the glycolysis process in hemocytes responded to pathogen infection differently between *V. parahaemolyticus*-resistant and -susceptible shrimp.

### 3.4. Knockdown of PC Inhibited the Expression of Genes in the NF-κB Signaling Pathway

**Figure 7:**
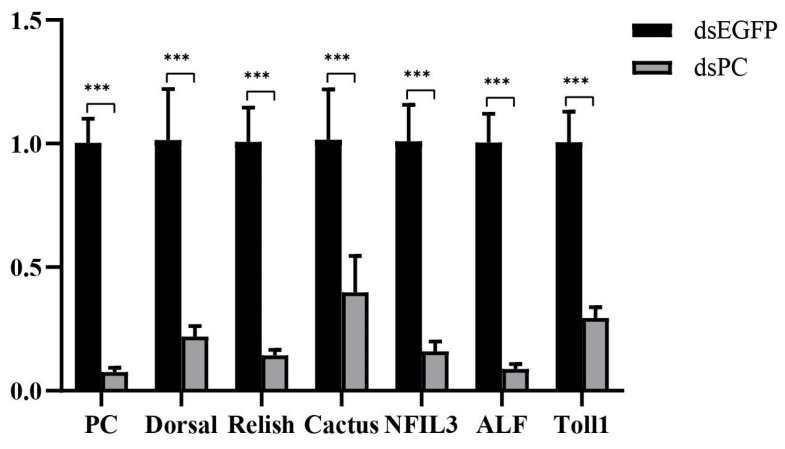
Expression changes of several genes in the NF-κB signaling pathway in hemocytes after PC knockdown. Significant differences between PC knockdown and control groups are shown with three asterisks at *p* < 0.001.

Previous study revealed that the down-regulation of PC could cause lactate accumulation, which then inhibited host immunity in mammals [3,15]. Therefore, PC was knocked down by RNAi, leading to 92% down-regulation of the gene (Figure 7). After PC knockdown, several genes in the NF-κB pathway, including Toll1, Doral, Relish, Cactus, nuclear factor interleukin 3 (NFIL3), and ALF, were all down-regulated by 71%, 78%, 86%, 61%, 84%, and 91%, respectively (Figure 7). The results suggested that the knockdown of PC led to the inhibition of the NF-κB signaling pathway in shrimp hemocytes.

## 4. Discussion

Shrimp usually have different resistance abilities against specific pathogens. In the present study, we found that although the main immune-related tissues, hemocytes, from shrimp with different resistances against *V. parahaemolyticus* have similar transcriptional profiles at the background level, it showed apparently different immune responses at the early infection stage. As the bacterial loads remained at a low level in all tested shrimp at 3 hpi, the differences in immune response should reflect the genetic variation among individuals rather than those caused by different proliferation speeds of *Vibrio* in shrimp. Therefore, different immune responses in the tested shrimp contributed to their distinct resistance against *V. parahaemolyticus* infection. However, it still needs to be verified in more groups of shrimps whether this phenomenon is generally accepted.

In the *V. parahaemolyticus*-susceptible shrimp, the immune response genes in hemocytes were mainly related to metabolic processes. This was similar to our previous reports on the hepatopancreas of *V. parahaemolyticus*-susceptible shrimp, in which many metabolic processes were responsive to *Vibrio* infection [8]. During infection, bacteria could manipulate host metabolism for their survival and replication. In the mice macrophages, *Salmonella typhimurium* promoted its intracellular replication through the up-regulation of host glycolysis and a decrease in host serine synthesis process, which would provide carbon sources for bacteria and increase the production of bacterial virulence factors [17]. Host lipids can be hijacked by bacteria to meet their energy needs or to hide themselves from the host cell [18]. Notably, the potential regulatory tandem of inflammatory response and amino acid metabolism, such as the biosynthesis of phenylalanine, tyrosine, and tryptophan and the metabolism of phenylalanine, has important functions in adaptive and innate immunity [19]. In *V. parahaemolyticus*-susceptible shrimp, there were significantly fewer immune-related genes in hemocytes responsive to *Vibrio* infection when compared with those in *V. parahaemolyticus*-resistant shrimp. These data suggested that changes in metabolic processes in *V. parahaemolyticus*-susceptible shrimp might reduce the resistance ability of *V. parahaemolyticus*-susceptible shrimp to the pathogen. However, the underlying mechanism needs to be further investigated.

In the hemocytes of *V. parahaemolyticus*-resistant shrimp, there were many more immune-related genes responsive to *Vibrio* infection. A comparative transcriptome analysis of the hepatopancreas from *V. parahaemolyticus*-resistant and -susceptible shrimp showed that, at the background level, the immune-related genes were highly expressed in *V. parahaemolyticus*-resistant shrimp and the metabolism-related genes were highly expressed in *V. parahaemolyticus*-susceptible shrimp, and the high expression profile was maintained after *Vibrio* infection [8]. Although there were few DEGs in hemocytes between *V. parahaemolyticus*-resistant and -susceptible shrimp at the background level, the immune responsive genes in hemocytes at 3 hpi exhibited similar characteristics as in the hepatopancreas. In the hemocytes of *V. parahaemolyticus*-resistant shrimp, some immune-related genes such as ankyrin repeat domain-containing protein, ficolin-1, venom protease, etc. were up-regulated after *Vibrio* infection. Previous studies have shown that these genes positively regulate host immunity [20,21,22]. Therefore, expression changes in the immune-related genes in the hemocytes after *Vibrio* infection might contribute to shrimp resistance ability to *V. parahaemolyticus*.

Some metabolism-related genes were also responsive to the infection in the hemocytes of *V. parahaemolyticus*-resistant shrimp. Among them, several glycolysis-related genes including GLUT2, HK, FBP, and PCK1 were all down-regulated after *Vibrio* infection. In the *V. parahaemolyticus*-resistant shrimp, the down-regulation of GLUT2 and HK could limit the cell uptake of glucose and its inflow into the process of glycolysis, which could reduce the level of glycolysis. In arthropods, trehalose acts as the principal hemolymph sugar, which can be degraded into glucose [23,24,25]. In the hemocytes of *V. parahaemolyticus*-resistant shrimp, a gene encoding trehalose transporter was also down-regulated after *Vibrio* infection, which would also lead to the low level of cellular glucose. Meanwhile, the down-regulation of FBP and PCK1 can limit the gluconeogenesis process, which promotes glucose entering glycolysis to effectively maintain the normal energy supply through glycolysis and the TCA cycle.

In contrast, the expression level of these genes was not affected by *Vibrio* in the hemocytes of the *V. parahaemolyticus*-susceptible shrimp. This could allow sufficient glucose to enter the glycolytic process. Meanwhile, the expression of pyruvate carboxylase (PC) was significantly down-regulated, which may inhibit the TCA cycle and cause lactate accumulation [26]. In mammals, lactate is regarded as a negative regulator of RLR signaling pathway and many important immune cells [5]. In the present study, we found that the knockdown of PC, which might lead to lactate accumulation, suppressed the expression of genes in the classical NF-κB signaling pathway. Notably, as an inhibitor of the NF-κB signaling pathway, the expression of Cactus was also inhibited after PC knockdown. This might be due to the knockdown of PC that can cause the overall inhibition of the NF-κB signaling pathway. It indicated that the host immune defense might be decreased under this condition. Therefore, the differential responses of genes in the glycolytic process in hemocytes might partially contribute to the distinct resistance abilities of shrimp to *V. parahaemolyticus*. Further investigation will be conducted to reveal the underlying regulatory mechanism.

## 5. Conclusions

In the present study, we illustrated that the immune responses in the hemocytes of shrimp with different *V. parahaemolyticus* resistance abilities vary greatly. The metabolic process, glycolysis, might affect host resistance to a *Vibrio* infection by directly influencing host immunity. The present data provide new insights into understanding the genetic mechanisms of shrimp disease resistance.

## Data Availability

The original contributions presented in the study are included in the article/Supplementary Material. Further inquiries can be directed to the corresponding authors.

## Supplementary Materials

The following supporting information can be downloaded at: https://www.mdpi.com/article/10.3390/biology13050300/s1, Table S1: Primers used in the present study, Table S2: Differentially expressed genes identified in L0h vs. D0h comparison, L0h vs. L3h comparison, and D0h vs. D3h comparison.
